# Supplementary material for: Products of Bisphenol A Degradation Induce Cytotoxicity in Human Erythrocytes (In Vitro)
Source: Int J Mol Sci. 2022 Dec 28;24(1):492. doi: 10.3390/ijms24010492 (PMC9820436; doi:10.3390/ijms24010492)
Supplement: Supplementary file 1 [file ijms-24-00492-s001.zip › ijms-2034363-supplementary.pdf]

International Journal of
*Molecular Sciences*

MDPI

# Supplementary materials.

# Products of bisphenol A degradation induce cytotoxicity in human erythrocytes (*in vitro*)

**Katerina Makarova [1,#], Ewa Olchowik-Grabarek [2], Krzysztof Drabikowski[3], Justyna Kurkowiak[1], and Katarzyna Zawada[1]**

[1] Department of Organic and Physical Chemistry, Faculty of Pharmacy, The Medical University of Warsaw, Banacha 1, 02-097 Warsaw, Poland
[2] Laboratory of Molecular Biophysics, Department of Microbiology and Biotechnology, Faculty of Biology, University of Bialystok, Konstanty Ciolkowskiego 1J, 15-245 Bialystok, Poland
[3] Laboratory of Biological Chemistry of Metal Ions, Institute of Biochemistry and Biophysics Polish Academy of Sciences, Pawińskiego 5a, 02-106 Warsaw, Poland
* Correspondence: kmakarova@wum.edu.pl

## 1. Toxicity studies on N2 worms

N2 worms is commonly used to study toxicity of heavy metals, various environmental pollutants, including pesticides and neurotoxins, for example bisphenol A. No data about phenol, hydroquinone or 4-isopropylphenol were reported. Previous studies reported effects of bisphenol A on the development of *C.elegans* rather at higher concentrations of bisphenols (228mg/L), causing abnormalities in mitochondria, short organismal lifespan, age-related changes in neurons, decrease ROS and increase ATP levels. [1] Here we did not observe any mortality of the nematodes in the range of concentrations 5-160mg/L.

***Oxidative stress***

To get information about oxidative stress induced by bisphenol A, phenol, hydroquinone or 4-isopropylphenol on C.elegans, we used transgenic worms that express the biosensors HyPer. Previously, it was shown that oxidized-to-reduced ratios of HyPer can be used as proxi for $H_2O_2$ level *in vivo*. [2] None of the compounds in the studied range of concentrations caused significant changes in oxidized-to-reduced ratios of HyPer (Fig.1)

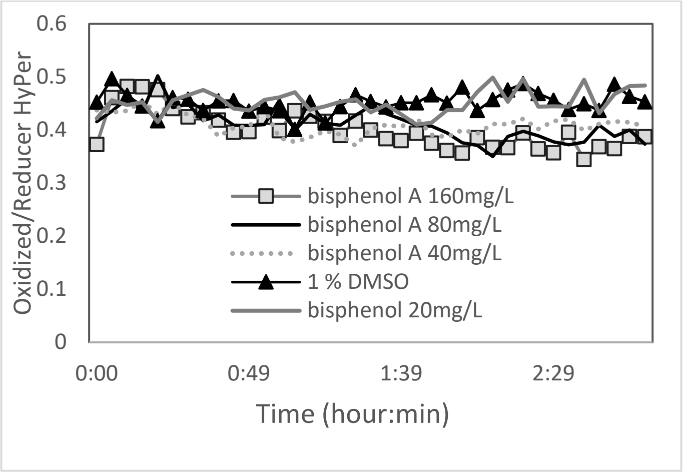


A

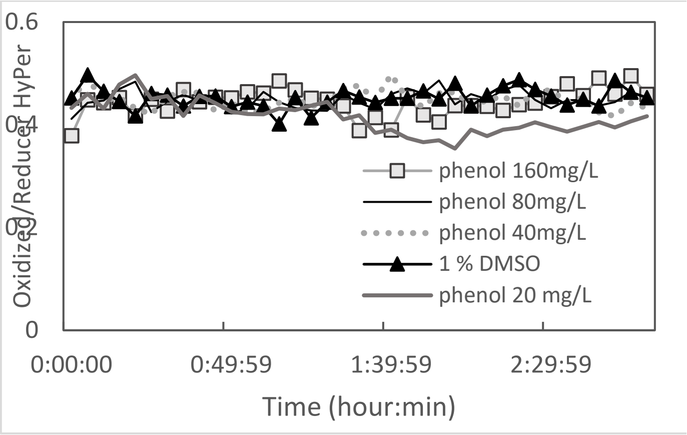


B

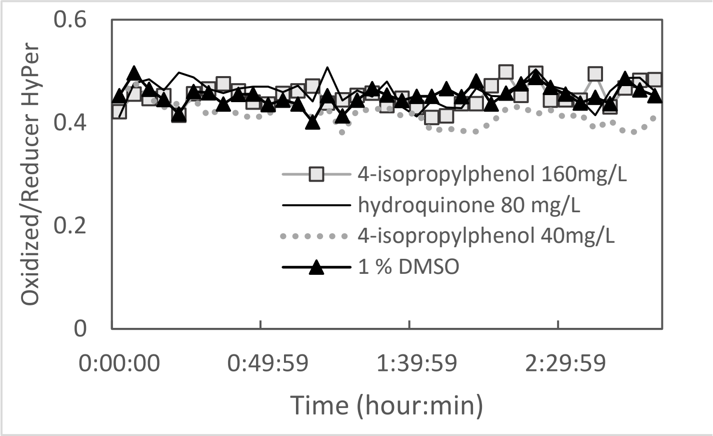


C

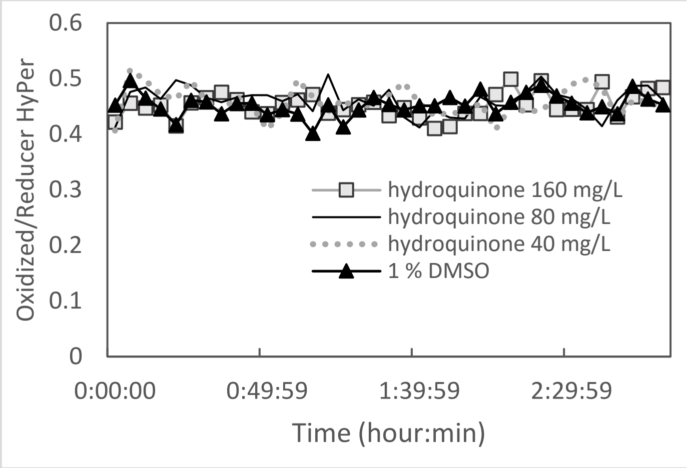


D

**Figure S1** In vivo HyPer response to (a) BPA; (b) phenol; (c) 4-isoprophylphenol; (d) hydroquinone treatment. Treatment with the selected compounds induced a transient dose-dependent increase in the ratio of oxidized to reduced HyPer in transgenic worms. The fluorescent transgenes were excited at 405 (reduced HyPer) and 490 nm (oxidized HyPer), and emission at 535 nm was quantified in a spectrofluorometer.

***Locomotive activity***

The movements of nematodes were disturbed, indicating neurotoxicity. Among studied compounds (Fig. S1) 4-isoprophylphenol causes changes in *C. elegans* locomotion pattern. Increased oscillating values suggests that the compound irritates N2 worms and is slightly neurotoxic

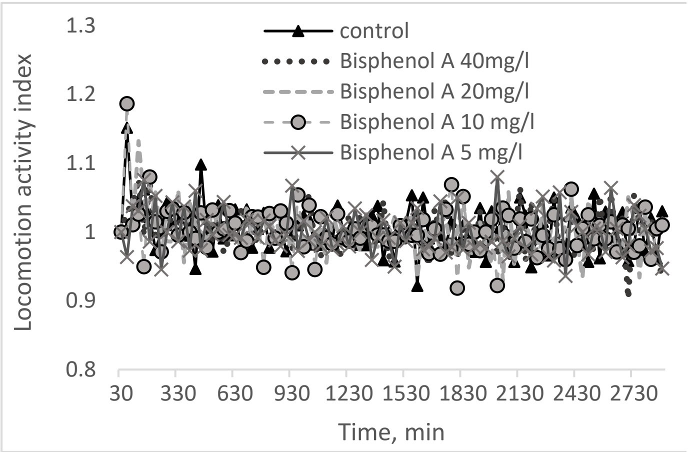


A

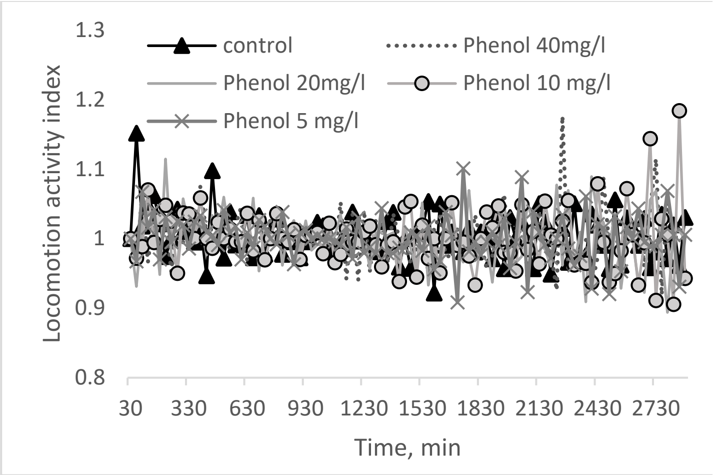


B

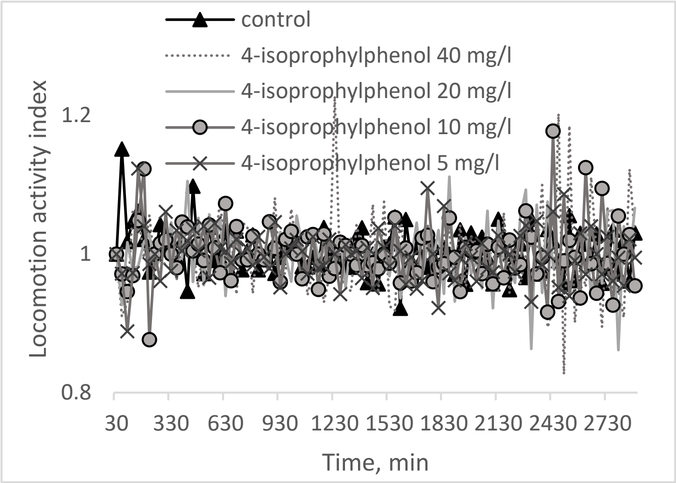


C

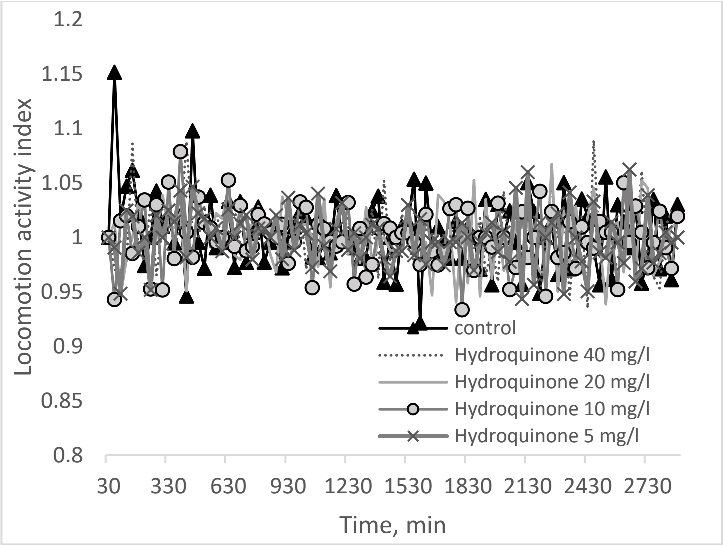


D

**Figure S2.** Normalized locomotive activity **for N2 worms exposed to** (a) BPA; (b) phenol; (c) 4-isoprophylphenol; (d) hydroquinone

## 2. Materials and Methods

*C.elegans* N2 (Bristol) worms were cultured according to standard procedures [3]. For neurotoxicity assessment worms were washed 3 times with K-medium (2.36 g KCl, 3 g NaCl, in 1 L dH2O). Approximately 100 mix stage worms were placed in 100 microL of K-medium per 96 well plate. The overall locomotion activity was assessed in Worm MicroTracker (InVivo Biosystems, OR, US). The data were integrated every 30 min during 48 hours. The concetration range from 5 to 40mg/L was used.

Construction of HyPer transgenic strain and spectrofluorometry measurements were performed as described elsewhere. [2]

## References

1. Hyun M, Rathor L, Kim H-J, et al.: Comparative toxicities of BPA, BPS, BPF, and TMBPF in the nematode Caenorhabditis elegans and mammalian fibroblast cells. *Toxicology.* 2021, *461:*152924.

2. Back P, De Vos WH, Depuydt GG, et al.: Exploring real-time in vivo redox biology of developing and aging Caenorhabditis elegans. *Free Radical Biology and Medicine.* 2012, *52:*850-859.

3. Brenner S. The genetics of Caenorhabditis elegans. *Genetics*. 1974 May;77(1):71-94.
